# Envenomation caused by *Rhopalurus amazonicus* Lourenço, 1986 (Scorpiones, Buthidae) in Pará State, Brazil

**DOI:** 10.1186/1678-9199-20-52

**Published:** 2014-12-09

**Authors:** Deyanira Fuentes-Silva, Alfredo P Santos-Jr, Joacir Stolarz Oliveira

**Affiliations:** Laboratory of Chemistry and Biological Macromolecule Structure, Federal University of Western Pará (UFOPA), Santarém, Pará State Brazil; Laboratory of Ecology and Animal Behavior, Federal University of Western Pará (UFOPA), Santarém, Pará State Brazil; Laboratory of Physiology and Animal Toxins (Fistox), Institute of Educational Sciences, Federal University of Western Pará (UFOPA), Av. Marechal Rondon, s/n, Caranazal, Santarém, Pará State CEP 68040-470 Brazil

**Keywords:** Scorpion sting, *Rhopalurus amazonicus*, Venomous animals, Envenomation, Brazilian Amazon, Pará

## Abstract

Scorpions, mainly those belonging to the genus *Tityus* cause many deaths and injuries in Brazil, with tens of thousands of envenomations notified every year. However, injuries involving other scorpion species are scarcely registered. Among the sixteen species of the genus *Rhopalurus*, Thorell, 1876, described up to date, nine are found in this country, with only a confirmed case of human envenomation provoked by *R. agamemnon* Koch, 1839. The present case reports, for the first time, a case of scorpion sting in a human victim involving *Rhopalurus amazonicus*, endemic species of the west region of the Pará state, Amazon, Brazil. The symptoms of envenomation were local pain and paresthesia. This study contributes to develop the knowledge on venomous scorpions, particularly those that may cause envenomations in this region.

## Background

It is known that scorpion envenomation comprises an important public health problem in Latin American countries, including Brazil, Cuba and Mexico [1–4]. According to the Brazilian Notifiable Diseases Information System (Sistema de Informação de Agravos de Notificação – SINAN), in 2013 almost eighty thousand cases of scorpion stings were registered in Brazil, which resulted in 75 deaths. To date, the states of Minas Gerais, São Paulo and Bahia have recorded the higher numbers of notifications concerning scorpion stings [5]. In those states, most envenomations are caused by *Tityus serrulatus* Lutz & Mello, 1922, *T. stigmurus* Thorell, 1876 and *T. bahiensis* Perty, 1833. These three species belong to the family Buthidae and their stings may provoke a broad range of clinical effects such as cardiotoxicity, neurotoxicity, respiratory dysfunction and, ultimately, lead to death [6, 7].

In northern Brazil, Pará is the state where scorpion stings are more frequent and the species *T. silvestris* and *T. obscurus* are responsible for the majority of cases. The latter, previously known as *T. cambridgei* or *T. paraensis*, is a big black scorpion, widely distributed in the Brazilian Amazon, particularly in Mato Grosso, Pará and Amapá states. It is considered the most important species responsible for human envenomations in those states [8–10].

According to the Brazilian Ministry of Health, the signs and symptoms of a scorpion sting may be classified as mild, with local pain and paresthesia; moderate, when there is sweating, nausea, occasional vomiting, tachypnea, tachycardia and mild hypertension; or severe when, in addition to the aforementioned symptoms, profuse and uncontrollable vomiting, intense sialorrhea, agitation, prostration, bradycardia, heart failure, pulmonary edema, shock, convulsions and coma are present. Death is attributed to the complications of pulmonary edema and shock [11]. These clinical manifestations fit in a more recent classification proposed by Khattabi *et al.*[12], in which mild, moderate and severe correspond respectively to Class I, characterized by local manifestations; Class II, that includes minor non-life-threatening manifestations; and Class III, when severe manifestations are observed, which at least include cardiac, respiratory or neurological failure.

The envenomation caused by *T. obscurus* frequently leads to local manifestations such as pain followed by paresthesia, edema, erythema, and, to a less extent, general systemic manifestations mainly consisting of sweating, agitation, tremors, nausea and myoclonus [8, 13]. Accidents involving other scorpion species are hardly notified, possibly because some victims develop only mild symptoms or healthcare services are not accessible by those who live in distant rural communities. Therefore, accidents involving different scorpion species, other than *T. obscurus*, are usually neglected or underreported cases.

**Figure 1:**
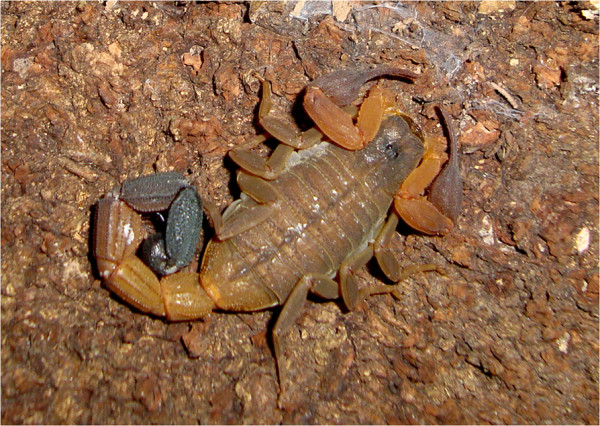
**Adult female of** ***Rophalurus amazonicus.***

Within of the Amazon forest, in Alter do Chão region, Pará state, the yellow scorpion *Rhopalurus amazonicus* is found, which also belongs to the family Buthidae (Figure 1). This endemic species was originally describe by Lourenço [14] and like other species of the genus *Rhopalurus*, it inhabits open Amazonian savannas, living under fallen tree trunks or under barks up to some meters above the ground. Furthermore, this species might be found inside houses.

According to Pardal *et al.*[8], the number of accidents provoked by the yellow scorpion in Santarém city, in the west of Pará state, was smaller than that caused by the species *T. obscurus.* Unfortunately, the authors did not mention the genus or the species of these yellow scorpions. This study is, to the best of our knowledge, the first description of an envenomation caused by the scorpion *R. amazonicus* in an adult man of 32 years who was handling these animals. The symptoms were local, around the sting area, and no other complications were observed.

## Case presentation

**Figure 2:**
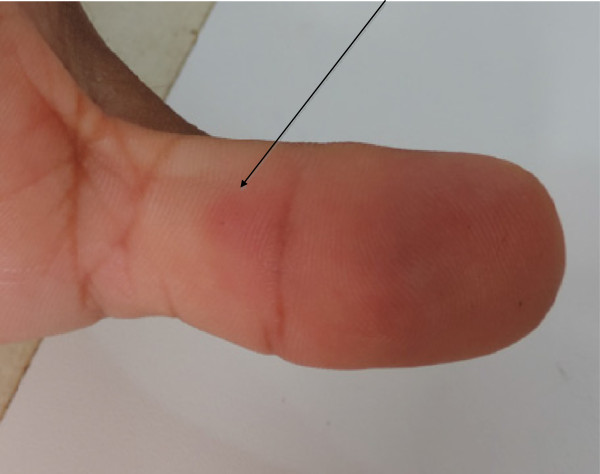
**Scorpion sting site on the middle of the thumb 56 hours after the accident.**

**Figure 3:**
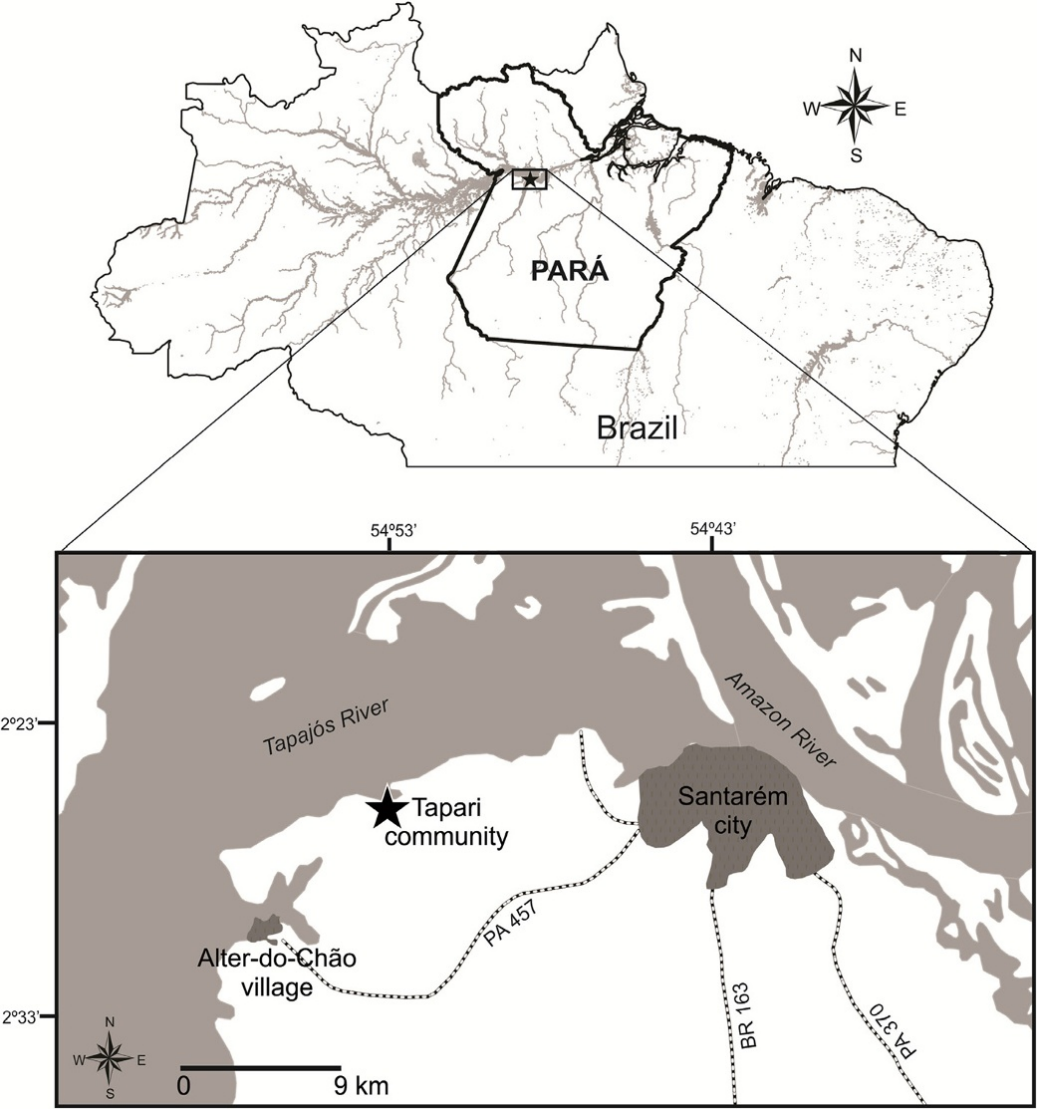
**Map of the Pará state, in the northern Brazil.** Inset emphasizes the hydrographic basin of the Tapajós river. The black star indicates Tapari, where the envenomation provoked by *R. amazonicus* occurred. Distance is shown on a relative scale bar. The map was created using the free software QGis 2.2.

A 32-year-old healthy male weighting 72 kg was stung by a scorpion on his right thumb while collecting these animals (Figure 2). The accident occurred in a savanna in the Tapari community, Santarém city, Pará state, Brazil (02° 26’ 54” S; 54° 53’ 25” W) on October 17, 2013 (Figure 3). Previously, the species was identified as *R. amazonicus* by the arachnologist who originally described it, Dr Wilson R. Lourenço [14].

The victim described local pain similar to a bee or wasp sting immediately after the scorpion sting, which rapidly spread throughout his arm, together with paresthesia, remarkably numbness and tingly sensations, 30 minutes after. Subsequently, he had a slight swelling that started in the thumb and spread to the arm during the first 12 hours along with severe itching that consisted of a crawling sensation on his skin from the hand to the arm. Hence, treatment with antivenom was not necessary. Approximately 56 hours after the accident, local and systemic symptoms disappeared.

## Discussion and conclusions

In Brazil, scorpion species of the genus *Tityus* are recognized as animals of medical importance, since they are responsible for high morbidity among adults as well as fatalities in children and the elderly. Studies on the symptoms and manifestations of envenomation caused by Brazilian *Tityus* are widely documented [1, 6–11, 13]. Nevertheless, accidents provoked by other species are rare or poorly reported in the literature, such as those caused by the genus *Rhopalurus*. Concerning this genus, up to date only three cases of human envenomation caused by *Rhopalurus agamemnon* have been described, all of them occurred in Piauí and Bahia states, in the Northeast region [15, 16]. Among them, only the case registered in Bahia had the species confirmed because it was collected. The clinical symptoms of envenomation by *R. agamemnon* were classified as a mild envenomation or Class I with paresthesia, shaking hands, tingling tongue, and curiously the victim did not present local pain [16]. In contrast, the main symptoms of the envenomation caused by *R. amazonicus* were radiating pain from sting site, paresthesia and severe itching sensation. Confirmed cases of envenomation by the yellow scorpion *R. amazonicus* have not been reported up to this moment. However, in 2003 Pardal *et al.*[8] mentioned the occurrence of accidents caused by yellow scorpions from the Amazon region of Santarém, but its scientific name was not provided.

Pará state records the highest number of scorpion stings in the North region of Brazil. According to SINAN [5], in 2013 the number of cases in this state was 1,885 (~54%) out of a total of 3,502 notifications registered for all seven states that comprise this region. In this concern, the notification of envenomation caused by venomous animals identified at least at the genus level still represents a gap in official data.

Herein, we presented the first report of an envenomation caused by *R. amazonicus*, an endemic species to the Amazon forest in Pará state, which, based on the symptoms, was classified as mild or Class I. However, it is known that symptoms provoked by envenomations depend on several factors such as the dose of venom injected, site of the sting, age, body mass and physiological conditions of the victim, being children and elderly the most vulnerable [5, 6, 9, 16, 17]. In conclusion, this report contributes to the knowledge on scorpion species capable of causing envenomations in Brazil. However, further study is required in order to evaluate the toxicity of this species on humans, which will make possible the classification of *R. amazonicus* as a scorpion of medical importance in the country*.*

## Consent

Written informed consent was obtained from the patient for publication of this case report and any accompanying images.
